# Critical maternal health knowledge gaps in low- and middle-income countries for the post-2015 era

**DOI:** 10.1186/s12978-015-0044-5

**Published:** 2015-06-05

**Authors:** Tamil Kendall, Ana Langer

**Affiliations:** Women and Health Initiative, Department of Global Health and Population, Harvard T.H. Chan School of Public Health, 651 Huntington Avenue, 7th Floor, Boston, MA 02115 USA

**Keywords:** Women’s health, Maternal health, Maternal mortality, Maternal morbidity, Research priorities, Global health, Low and middle-income countries (LMIC), Post-2015 development agenda, Implementation science, Implementation research

## Abstract

Effective interventions to promote maternal health and address obstetric complications exist, however 800 women die every day during pregnancy and childbirth from largely preventable causes and more than 90 % of these deaths occur in low and middle income countries (LMIC). In 2014, the Maternal Health Task Force consulted 26 global maternal health researchers to identify persistent and critical knowledge gaps to be filled to reduce maternal morbidity and mortality and improve maternal health. The vision of maternal health articulated was comprehensive and priorities for knowledge generation encompassed improving the availability, accessibility, acceptability, and quality of institutional labor and delivery services and other effective interventions, such as contraception and safe abortion services. Respondents emphasized the need for health systems research to identify models that can deliver what is known to be effective to prevent and treat the main causes of maternal death at scale in different contexts and to sustain coverage and quality over time. Researchers also emphasized the development of tools to measure quality of care and promote ongoing quality improvement at the facility, district, and national level. Knowledge generation to improve distribution and retention of healthcare workers, facilitate task shifting, develop and evaluate training models to improve “hands-on” skills and promote evidence-based practice, and increase managerial capacity at different levels of the health system were also prioritized. Interviewees noted that attitudes, behavior, and power relationships between health professionals and within institutions must be transformed to achieve coverage of high-quality maternal health services in LMIC. The increasing burden of non-communicable diseases, urbanization, and the persistence of social and economic inequality were identified as emerging challenges that require knowledge generation to improve health system responses and evaluate progress. Respondents emphasized evaluating effectiveness, feasibility, and equity impacts of health system interventions. A prominent role for implementation science, evidence for policy advocacy, and interdisciplinary collaboration were identified as critical areas for knowledge generation to improve maternal health in the post-2015 era.

**Electronic supplementary material:**

The online version of this article (doi:10.1186/s12978-015-0044-5) contains supplementary material, which is available to authorized users.

## Background

Despite sustained and focused efforts to reduce maternal mortality and the existence of effective interventions to promote maternal health and address obstetric complications, [1, 2] 800 women die every day during pregnancy and childbirth from largely preventable causes. More than 90 % of these deaths occur in low and middle income countries (LMIC) [3]. In 2000, as part of the Millennium Development Goals, United Nations member states committed to reducing maternal mortality by three-quarters by the end of the 2015. It is now clear that only a small number of countries will achieve this goal, [4] and that sustained commitment to improving maternal health must be central to the post-2015 development agenda. Urgent action is needed to identify the most pressing evidence gaps that are contributing to limited progress in the countries with the highest burden of maternal morbidity and mortality. Between June and October 2014, the Maternal Health Task Force at the Harvard T.H. Chan School of Public Health consulted 26 international maternal health researchers about priorities for knowledge generation to improve maternal health as we evaluate progress and look towards the post-2015 development agenda [5]. The acceptance rate to our invitation was overwhelmingly positive (87 %), which we interpreted as an indication of the perceived urgency of this discussion among the maternal health researchers’ community. Respondents were asked open-ended questions to identify the persistent and critical knowledge gaps that need to be filled to improve maternal health outcomes and reduce maternal mortality and morbidity in LMIC; crucial maternal health issues that have not been given adequate attention by researchers and donors; and new situations and emerging challenges that require research, with a special focus on implementation [6]. A summary of the content analysis is provided in Table 1 and the full working paper is available at www.mhtf.org [7]. In this commentary, we share the priorities that were identified by this group of outstanding researchers, in an effort to stimulate further reflection and discussion about these critical topics within the global maternal health community.

**Table 1:** Content analysis of identified priorities for knowledge generation to improve maternal health in low- and middle-income countries^a^

N = 26 respondents	Number of respondents who identified the topic as a priority
Persistent and critical maternal health knowledge gaps	
Implementation research to strengthen health systems to deliver evidence-based interventions at scale and with quality	19
Improving quality of maternal healthcare	16
Improving the quality and availability of information about maternal mortality	7
Supporting women’s empowerment	7
Increasing the availability and uptake of contraception	6
Increasing access to safe abortion services	6
New treatments for the major causes of maternal death	6
Crucial maternal health issues that have not received adequate attention from donors and researchers	
Health workforce (allocation and retention, task-shifting, training, and leadership and supervision; role of private and unrecognized providers)	15
Preventing and eliminating disrespect and abuse	9
Over-medicalization of birth	8
Demand generation	7
Measurement, prevention, and treatment of maternal morbidities	7
New situations and emerging challenges that affect maternal health	
Increasing burden of non-communicable disease	13
Persistence of social and economic inequality	10
Urbanization	9
Translating knowledge about the developmental origins of health and disease into practice	7
Information and communication technologies to enhance maternal health	7

^a^Numbers sum to more than 26 as respondents were not limited in the number of priority topics for knowledge generation that they could mention. There was significant overlap in the topics identified by respondents as “persistent knowledge gaps” and those considered “not to have received adequate attention from donors and researchers”. The decision to classify a specific topic as a “persistent knowledge gap” or an area that has “not received adequate attention” was made based on: a) the frequency with which a topic was mentioned in response to one or the other question and b) grouping of related topics

### Critical maternal health knowledge gaps

#### Persistent and critical maternal health knowledge gaps

Respondents emphasized that efforts to generate new knowledge should focus on strategies to strengthen health systems and improve the quality and reach of service delivery. This point was summed up by the researcher who said: *“We know what to do. But the interactions between the interventions and the health system have not been studied.”* Health systems research questions mentioned during the interviews included identifying models that can deliver what is known to be effective to prevent and treat the main causes of maternal death at scale in different contexts, and to sustain coverage and quality over time. The second most commonly mentioned priority area for knowledge generation was improvement of quality of care. In many settings, particularly in Asia, the proportion of women giving birth in facilities is rapidly increasing [8], but respondents commented that *“coverage alone isn’t sufficient to save lives and guarantee human rights”* and expressed concern that *“we have no idea about the quality of services that we are pushing women into”.* Information about quality of care at the national, district and facility level is needed. Respondents recommended participatory approaches for monitoring quality and quality improvement, and prioritized refinement of quality of care indicators and development of easy to use tools to facilitate data collection. Other specific topics mentioned frequently were increasing the availability and quality of information about maternal mortality, particularly by improving vital registration systems; evaluating how interventions to empower women as healthcare users and healthcare providers affect health system functioning and health outcomes; and knowledge generation to support policy advocacy to increase the availability, accessibility, acceptability, and quality of essential services, including contraception and safe abortion services.

#### Crucial issues in maternal health that have not received adequate attention from donors and researchers

The health workforce was the most frequently mentioned topic considered not to have received adequate attention from donors and researchers. Respondents identified the need for implementation research to improve distribution and retention of healthcare workers; facilitate task shifting; and develop and test innovative training approaches to improve “hands-on” skills, promote evidence-based practice, produce attitudinal and behavioral changes, and increase managerial capacity at different levels of the health system. Many respondents noted the need to reorganize the health workforce, and related need to transform power relationships within institutions and between groups of healthcare providers. One interviewee stated that this transformation would require *“profound changes in how institutions are structured and how the actors in institutions relate to each other.”* Research is needed to identify how to effectively bring about changes in disciplinary and institutional hierarchies. Other specific topics frequently mentioned as having been relatively neglected by researchers and donors were certain aspects of clinical practice, in particular over-medicalization of birth, specifically rising rates of unnecessary caesarean deliveries as more women give birth in facilities [9]; prevention and elimination of disrespect and abuse during maternity care; and the measurement, prevention, and treatment of maternal morbidities.

#### New situations and emerging challenges that affect maternal health

Respondents identified the following as the key factors that will shape the landscape of maternal health over the next decade and require knowledge generation to improve outcomes: increasing burden of non-communicable diseases among pregnant women and women of reproductive age; maternal health challenges in urban settings; and the measurement of persistent social and economic inequalities. In particular, respondents stated that vulnerable groups and “*the lowest quintiles are not going away and the situation may become relatively worse for them.”* They emphasized the scarcity of data and indicators to measure social and economic inequality, and commented that existing metrics are narrowly focused on economic inequality. Respondents called for development of more sophisticated measures of inequality to explain variation in quality of care and health outcomes and to track progress on reducing equity gaps.

With respect to new opportunities, the most frequently mentioned was the potential for information and communication technologies to enhance decision-making by women, healthcare providers and policymakers. Several respondents identified the need to translate the growing evidence about the developmental origins of health and disease, specifically how women’s health prior to conception and the uterine environment influence the long-term health outcomes of their children, into policy, programs and measurement through health information systems. The need to attend to geopolitical determinants of maternal health, such as climate change, food insecurity, the proliferation of conflict and humanitarian crises, and the rise of religious fundamentalism, was also mentioned.

#### Research approaches

While respondents were not specifically asked about research methods, the urgent need for well-designed implementation research and evaluation was a recurrent theme. Insistence on the importance of re-orientating knowledge generation was summed up by the respondent who stated: “*There has been more focus on the drugs and the magic bullets, there has been a lot of focus on the interventions and inventions—the need [now] is to focus less on the inventions and more on implementation research.”* Respondents also expressed that persuading donors of the value of rigorous evaluation was necessary to advance maternal health, noting that *“when donors think about adding value, they do not think about evaluation. We as researchers need to change that conversation.”*

Respondents stressed the need to think strategically about the relationships between knowledge generation and policy advocacy as we move into the post-2015 era. They identified policymakers’ lack of knowledge about public health and maternal health as critical barriers to the development of evidence-informed policy and allocation of funds for maternal health. Proposed solutions included providing basic public health education to policymakers, increasing the research capacity of national Ministries of Health, and engaging policymakers in the development and implementation of maternal health research. Further, respondents emphasized the need for *“research as advocacy, research for political argumentation”* to advance implementation of what works, particularly for controversial topics (*e.g.* medical abortion). National demonstration projects and estimates of costs, lives saved, and disabilities averted were mentioned as promising evidence to support policy advocacy.

Finally, the need to overcome disciplinary siloes within maternal health was frequently mentioned. Respondents identified that going beyond narrowly defined areas of specialization would advance maternal health: *“I am a pre-eclampsia-ologist, there are postpartum hemorrhage-ologists—everyone is an ologist, we need maternal health-ologists to pull it all together.”* Respondents also made emphatic and repeated calls for interdisciplinary research, particularly the need to collaborate with social scientists.

## Conclusions

The global maternal health researchers consulted by the Maternal Health Task Force placed high priority on implementation research to improve the delivery of existing evidence-based maternal health interventions, echoing the results of a recently published international survey on priorities for maternal and perinatal health research [10]. The vision of maternal health shared by respondents, and the corresponding priorities to improve outcomes for women, were comprehensive and surprisingly consistent among the wide range of researchers we interviewed. Priorities for knowledge generation encompassed improving the availability, accessibility, acceptability, and quality of labor and delivery services and other evidence-based interventions, such as contraception and safe abortion services. Respondents emphasized the importance of health systems research to identify models that can deliver what is known to be effective to prevent and treat the main causes of maternal death at scale in different contexts and to sustain coverage and quality over time, and the development of tools to measure quality of care and promote ongoing quality improvement at the facility, district, and national level. The healthcare workforce was the most frequently mentioned critical topic perceived to have been neglected by researchers and donors. Respondents noted that attitudes, behavior, and power relationships between health professionals and within institutions must be transformed to achieve coverage of high-quality maternal health services in LMIC, and that research could provide insights on how to do it. Respondents identified the increasing burden of non-communicable diseases, urbanization, and the persistence of social and economic inequality as emerging challenges that require knowledge generation to improve health system responses and evaluate progress. Effectiveness, feasibility, and equity impacts of health system interventions should be evaluated. Specific topics mentioned by respondents as priority areas for knowledge generation are provided as Additional file 1. While the consultation did not ask specifically about research approaches, respondents highlighted the importance of greater prominence for implementation science, evidence for policy advocacy, and interdisciplinary collaborations as critical areas for knowledge generation to improve maternal health post-2015.

## Additional file

Additional file 1:
**Summary of specific topics for knowledge generation to improve maternal health in low- and middle-income countries mentioned by respondents.**
