# Supplementary material for: Critical maternal health knowledge gaps in low- and middle-income countries for the post-2015 era
Source: Reprod Health. 2015 Jun 5;12:55. doi: 10.1186/s12978-015-0044-5 (PMC4475304; doi:10.1186/s12978-015-0044-5)
Supplement: Additional file 1: — Summary of specific topics for knowledge generation to improve maternal health in low- and middle-income countries mentioned by respondents. [file 12978_2015_44_MOESM1_ESM.docx]

**Summary of specific topics for knowledge generation to improve maternal health in low- and middle-income countries mentioned by respondents**

| **PERSISTENT AND CRITICAL MATERNAL HEALTH KNOWLEDGE GAPS: SPECIFIC TOPICS** |
| --- |
| **Implementation research to strengthen health systems to deliver evidence-based interventions at scale and with quality**   - How to translate the known evidence base for prevention and treatment of maternal mortality into large scale actions of high quality and sustain implementation in different contexts? - What is the portfolio of key interventions (technical interventions and health system reforms) to improve maternal health in different settings? - Model of care: what interventions should be delivered by different human resources and where? - Implementation research on interventions to overcome geographic barriers to women accessing comprehensive emergency obstetric services (transportation, maternity waiting homes). |
| **Improving quality of maternal healthcare**   - Landscape analysis of interventions shown to improve quality of care. - Documentation of service operation and coverage, and analysis of related health outcomes. - Refinement of measures of facility readiness. - Validation of indicators to estimate coverage of interventions (uterotonics, breastfeeding, postpartum checkup). - Development of a simple “checklist” or “dashboard” to evaluate quality of care that can be used for frequent monitoring at the facility level, and evaluation of implementation. - Development of health information systems that link measures of quality to health outcomes, including in the community during the postpartum period. |
| **Improvement of the availability and quality of information about maternal mortality**   - What types of implementation research and policy advocacy improve vital registration? - How does strengthening death audits at the country and facility level affect maternal health? - Improvement of information about causes of maternal death in community (improvement of verbal autopsies, implementation research on minimally invasive autopsies). - Improved measurement of indirect causes of maternal death, and analysis of the relationships between direct and indirect causes of maternal death. |
| **Supporting women’s empowerment**   - Evaluation of the effects of interventions to improve women’s status, autonomy, decision-making, and control over resources on maternal health. These could include economic empowerment (e.g. cash transfer, vouchers, access to mobile banking), community-based initiatives to improve quality and reach of maternal healthcare (e.g. women’s groups), and transformation of gender norms to increase women’s freedom of movement and reduce violence against users and providers of health services. - Evaluation of social accountability and participation mechanisms on coverage and quality of care and health outcomes. |
| **Increasing availability and uptake of contraception**   - What are effective models for increasing use of contraceptives, particularly long acting reversible contraceptives (LARC), while ensuring respect for women’s rights and choice? - Identification of women’s knowledge/beliefs and preferences, as well as providers’ knowledge of, beliefs about and skills to provide LARC. |
| **Increasing access to safe abortion services**   - What are the policy and service delivery barriers to implementing the best practices to increase access to safe abortion services, including medical abortion? - How can task-shifting that allows providers other than physicians to provide abortion services be implemented in different settings? - How do we overcome stigma and inequities in access to safe abortion services? |
| **New treatments for major causes of maternal death**   - What are effective alternative methods of treating pre-eclampsia and eclampsia and obstetric hemorrhage? - What are the causes of hypertension during pregnancy and how can we treatment them? |
| **CRUCIAL MATERNAL HEALTH ISSUES THAT HAVE NOT RECEIVED ADEQUATE ATTENTION FROM DONORS AND RESEARCHERS: SPECIFIC TOPICS** |
| **Health Workforce**   - What financial and non-financial incentives are effective for improving healthcare worker distribution, retention, and performance? - Programs that train local people to provide maternity services should be rigorously evaluated. - Need to address gender discrimination to improve conditions for female healthcare workers (gender norms that limit women’s movement, workplace discrimination, etc.). - *Task-shifting* - How can political and regulatory barriers to task shifting be overcome? - Evaluation of provision of cesarean section and abortion services by non-physician health workers. - *Training* - Evaluation of alternative modes of acquiring hands-on skills (simulation, extending training beyond teaching hospitals). - Implementation research on interventions to improve and maintain skills (skills and drills, midwife exchange) and monitoring to evaluate use of skills learned in practice. - Knowledge generation to permit standardized measurement and clinical protocols for maternity care. - Evaluation of training that uses Information and Communication Technologies (ICT). - Implementation research on how to transform hierarchical relationships and training practices and promote evidence-based practice. - *Supervision and leadership* - Evaluation of training/creation of alternative cadres to increase managerial capacity. - Evaluation of ICT to support administration and provide tools for managerial decision-making - *Role of private/unregulated providers in health service delivery* - Assessment of services provided and women’s health outcomes. - What are effective models for training, accreditation and regulation of these providers? - How can governments effectively communicate the quality of care offered by private providers to women? |
| **Over-medicalization of birth, particularly unnecessary cesarean delivery**   - What health systems adjustments can reduce unnecessary caesarean deliveries? - How does introduction of ultrasound and electronic fetal monitoring in under-resourced health systems affect quality of care? |
| **Prevention and elimination of disrespect and abuse**   - What are the causes of disrespect and abuse? Need for both qualitative and quantitative research on provider behavior in rural and urban contexts. - What are the relationships between working conditions and disrespect and abuse? - Evaluate interventions designed to reduce disrespect and abuse (e.g. open birth days, pre-service and in-service training for healthcare providers, social accountability processes). - What are the barriers to implementation of continuous birth support? Does continuous birth support affect disrespect and abuse? |
| **Demand generation**   - What are the sociocultural barriers to women seeking facility-based birth in different African contexts? - How can we optimize the functioning of interventions to overcome financial barriers to utilization of maternal health services (cash transfers, vouchers, insurance for the poor)? - What are the effects of incentivizing antenatal care or postpartum care for women or providers? - Addressing the effects of elimination of user fees on facility level health financing. |
| **Measurement, prevention and treatment of maternal morbidities**   - Validation of maternal “near miss” and implementation research to promote mainstreaming. - Need to develop and standardize definitions of maternal morbidities and develop measurement tools. - Need for research on prevention of morbidities and implementation research on scale up of interventions to address anemia and fistula. - Analysis of the health system and broader social and economic costs of maternal morbidity and the cost-effectiveness of prevention and palliation. |
| **NEW SITUATIONS AND EMERGING CHALLENGES THAT AFFECT MATERNAL HEALTH:**  **SPECIFIC TOPICS** |
| **Increasing burden of non-communicable disease**   - Prevalence of obesity, diabetes, and hypertension among pregnant women in LMIC. - Development and evaluation of appropriate guidelines for prevention, screening, and management of women with chronic conditions during childbirth to improve outcomes. - Implementation research to evaluate models of service delivery for NCD as part of maternal healthcare and to strengthen referral systems. |
| **Persistence of social and economic inequality**   - Generation of more knowledge about social and economic inequality and development and implementation of metrics that go beyond measuring economic inequality. - Evaluation of equity enhancing interventions and differential programming. - *Populations specifically prioritized for attention included*: rural and urban poor; mobile populations (migrants, nomads, pastoralists); informal sector workers; those affected by religious fundamentalisms or residing in areas with marked gender inequality; individuals who experience infertility; and adolescents. |
| **Translating knowledge about the developmental origins of health and disease into practice**   - Development of health information systems to capture intergenerational outcomes. - Reconceptualization of maternal health to include women’s and girls’ health before conception (the preconception period and earlier). - Current evidence underlines the importance of prioritizing improvements in women and girls’ nutritional status and reducing exposure to environmental pollutants. |
| **Information and communication technologies (ICT) to enhance service delivery and utilization**   - Evaluate how ICT can increase access to information for women, providers, and healthcare decision-makers (managers). - Evaluate how ICT can empower participation and improve decision-making and service delivery and access for women, providers, and healthcare decision-makers. |
